# Tissue regenerative medicine: Clinical advances, challenges, and opportunities

**DOI:** 10.1063/5.0296897

**Published:** 2025-12-18

**Authors:** Seleem Badawy, Varshiny Gopinath, Ana M. Diaz Espinosa, Kasinan Suthiwanich, Ethan C. Kelmser, Victoria Duke, Veda Kamaraju, Karina Nakayama, Manoj Manna, Julianne J-Y Liu, Sara S. Nunes, Keyue Shen, Ngan F. Huang

**Affiliations:** 1 Department of Cardiothoracic Surgery, Stanford University, 300 Pasteur Drive, MC 5407, Stanford, California 94305, USA; 2 Center for Tissue Regeneration, Repair and Restoration, Veterans Affairs Palo Alto Health Care System, Palo Alto, California 94304, USA; 3 Department of Urology, University of California San Diego Medical Center, San Diego, California 92121, USA; 4 Department of Biomedical Engineering and Physiology, Mayo Clinic, Rochester, Minnesota 55905, USA; 5 Toronto General Hospital Research Institute, University Health Network, Toronto, Ontario M5G 2C4, Canada; 6 Department of Biomedical Engineering, Oregon Health & Science University, Portland, Oregon 97239, USA; 7 Department of Biomedical Engineering, Cornell University, Ithaca, New York 14853, USA; 8 Department of Materials Sciences, Stanford University, Stanford, California 94305, USA; 9 Institute of Biomedical Engineering, University of Toronto, Toronto, Ontario M5S 3G9, Canada and Laboratory of Medicine and Pathobiology, University of Toronto, Toronto, Ontario M5S 3K3, Canada; 10 Heart & Stroke/Richard Lewar Centre of Excellence, University of Toronto, Toronto, Ontario M5S 3H2, Canada; 11 Ajmera Transplant Center, University Health Network, Toronto, Ontario M5G 2N2, Canada; 12 Department of Biomedical Engineering, University of Southern California, Los Angeles, California 90089, USA; 13 Cardiovascular Institute, Stanford University, Stanford, California 94305, USA; 14 Geriatric Research, Education, and Clinical Center, Veterans Affairs Palo Alto Health Care System, Palo Alto, California 94304, USA

## Abstract

Regenerative medicine is transforming how we restore tissue function, leveraging advances in cell and molecular biology, biomaterials, and engineered microenvironments. While there have been notable advances and rapid progress over the past few decades, ongoing challenges persist in the technical development and effective translation of these advancements to clinical care. This perspective highlights clinically promising examples and critically assesses present challenges in translating tissue regenerative medicine therapies from the bench to the clinic. We further examine the evolving landscape of regenerative medicine by describing strategies to optimize the cellular microenvironment, the impact of patient demographics, and the use of artificial intelligence to shape the future of this field.

## INTRODUCTION

**FIG. 1.**
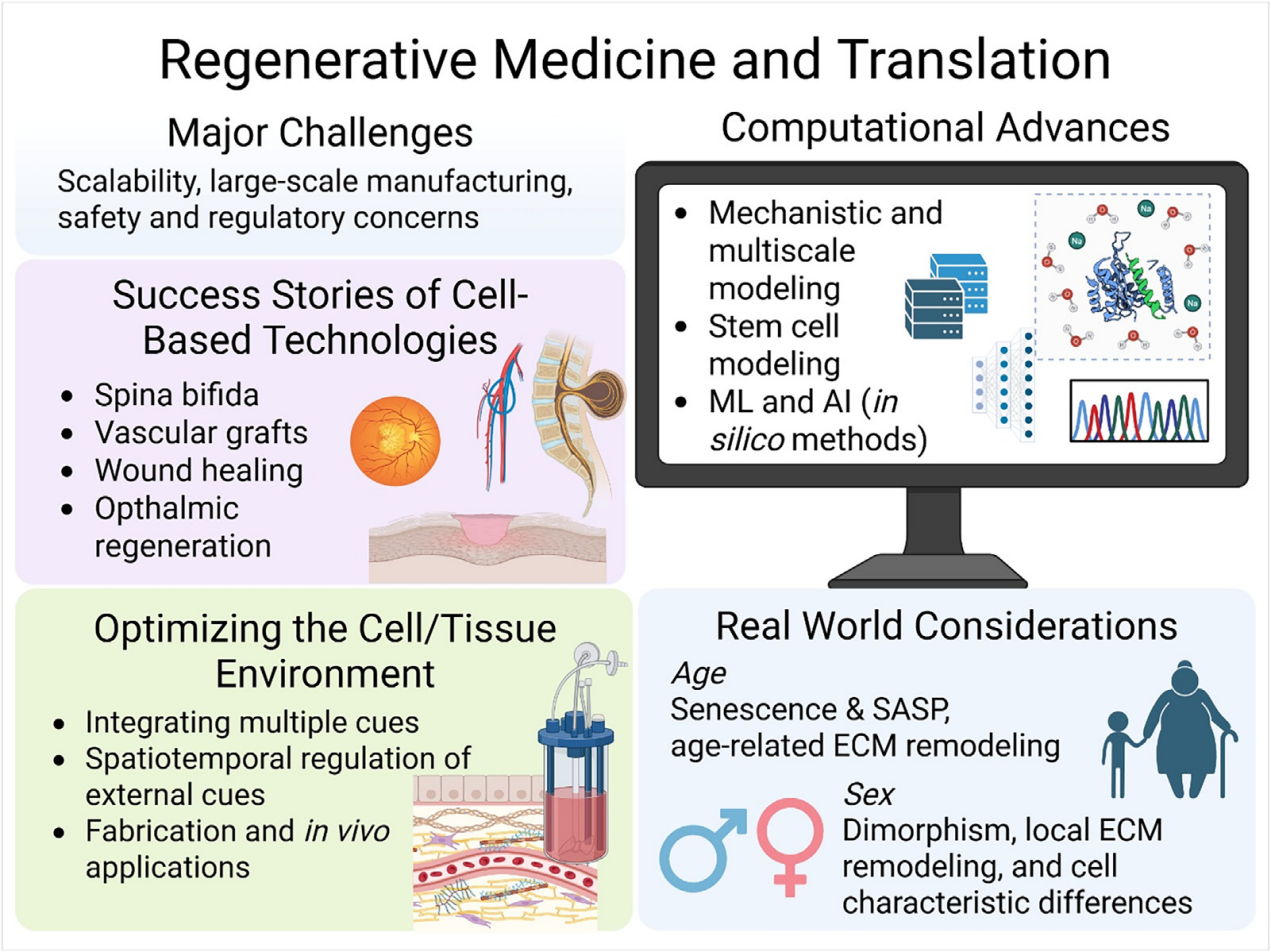
Schematic overview of emerging concepts and current themes across regenerative medicine. The figure includes the major challenges in clinical translation from bench to bedside of regenerative medicine technologies, success stories of cell-based technologies, optimizing the cell/tissue environment, real-world considerations, and computational advances. Abbreviations: ML, machine learning; AI, artificial intelligence; SASP, senescence-associated secretory phenotype; and ECM, extracellular matrix. Created in BioRender. Badawy, S. (2025) https://BioRender.com/30db3i5.

Regenerative medicine enables the repair or replacement of damaged tissues, thereby restoring physiological function.^1^ It is a rapidly advancing field with successful technologies that have achieved regulatory acceptance and public support for their unique potential to improve clinical outcomes. Despite this progress, regenerative medicine still faces technical limitations, such as optimizing the cell and tissue environment. Recent advances have enabled the development of increasingly complex models and engineered constructs that more closely mimic the native environment, from organoids and induced pluripotent stem cell (iPSC)-derived cells to bioengineered tissues and tunable scaffolds. Many promising technologies are tested in preclinical or clinical stages of development,^1^ but only a small fraction of novel therapies successfully navigate the path to clinical translation. Some of the challenges faced in regenerative medicine relate to technical scalability and the regulatory pathway. Moreover, biological variables such as age and sex^2^ remain understudied in both preclinical design and clinical implementation, limiting the ability for patient-specific customization and consistency of outcomes. This perspective article examines regenerative medicine and its translation from the laboratory setting to clinical application. Here, we discuss clinical successes, major translational challenges, real-world biological considerations such as age and sex, emerging strategies for optimizing the cell and tissue environment, and advances in computational modeling and artificial intelligence that together drive the development of regenerative medicine technologies toward patient care (Fig. 1).

## SUCCESS STORIES OF CELL-BASED TECHNOLOGIES FOR REGENERATIVE MEDICINE

Notable successes and recent innovations across human diseases illustrate the potential of cell-based engineering technologies for regenerative medicine. The first cell-based therapies for humans emerged in the 1950s in the form of bone marrow transplants for blood-borne cancers, with the first allogeneic transplantation performed in 1957.^3^ In addition, since gaining U.S. Food and Drug Administration (FDA) approval in the 2010s, CAR-T cell therapy has notably accelerated the commercialization of other cell-based therapies broadly.^4^ For example, in spina bifida, a spinal birth defect causing paralysis, hydrocephalus, and impaired urinary and digestive function, *in utero* cell therapies have been investigated to improve the ability to walk and other symptoms, along with notable surgical advancements in spina bifida care.^5^ The CuRe (Cellular Therapy for In Utero Repair of Myelomeningocele) Trial, which started in 2021 (ClinicalTrials.gov ID: NCT04652908), is the first FDA-approved clinical trial using placenta-derived mesenchymal stem cells for the treatment of myelomeningocele, the most severe form of spina bifida.^5,6^ Preclinical animal studies using these cells demonstrated significant improvements in motor function, particularly in walking, and the preservation of large neurons in lambs with spina bifida.^7^ With these strong results both *in vitro* and *in vivo,*^7,8^ the currently recruiting CuRe trial [as of April 2025 (Ref. 6)] has recently been awarded $15 × 10^6^ and holds strong potential to transform spina bifida care and improve patient quality of life.

**FIG. 2.**
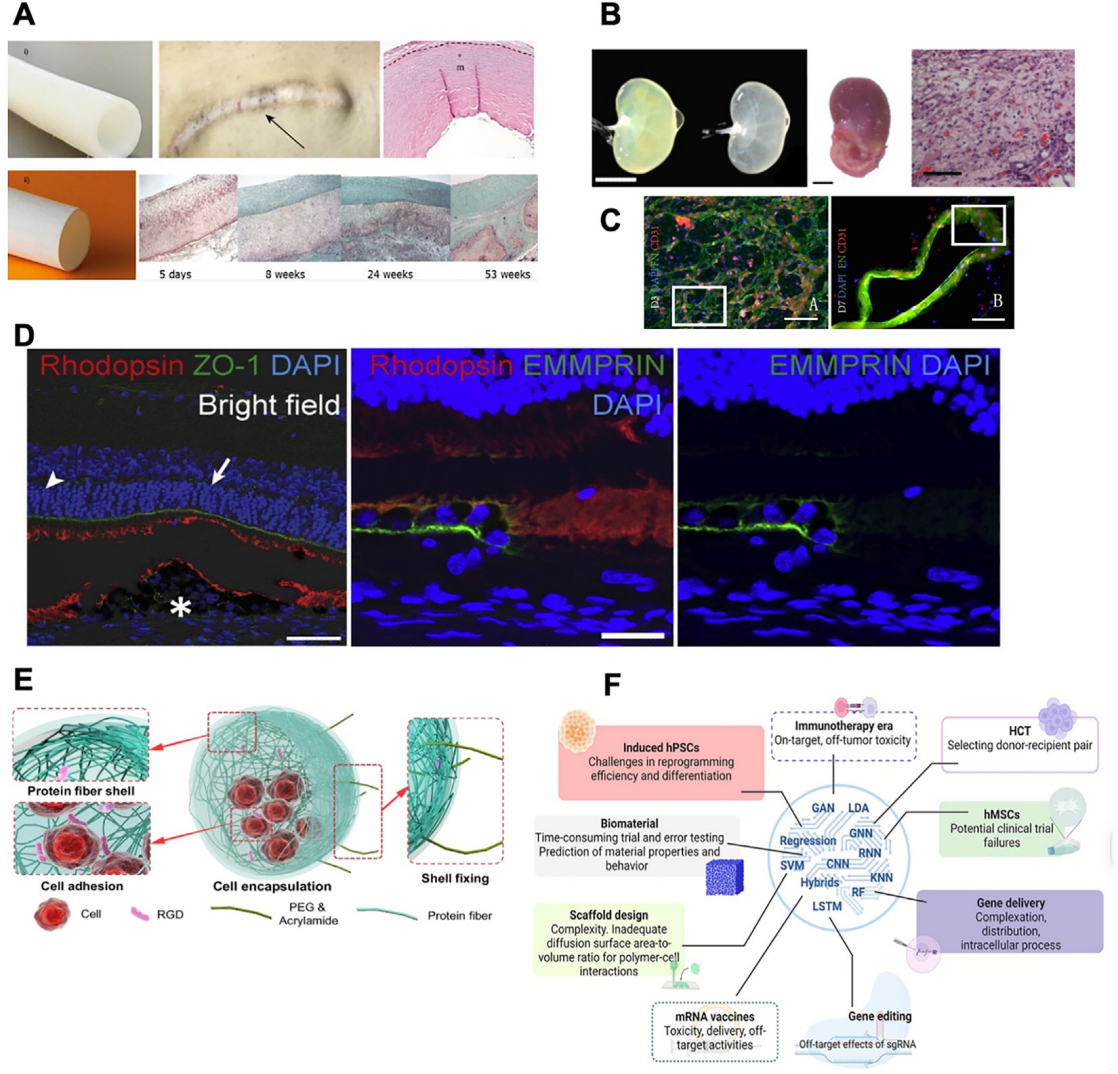
Panel highlights recent advances and translational potential of tissue engineering and regenerative medicine. (a) Study demonstrating the translational potential of tissue-engineered grafts: (i) An avascular graft for dialysis access developed by Humacyte showed biointegration and cell regeneration. (ii) A bioresorbable graft showed improved cavopulmonary conduit by improving remodeling.^11^ (b) Studies on regeneration potential of ECM-based scaffold: (i) transplantation of decellularized renal scaffolds alleviated nephrectomy and improved renal function, developed by Xeltis.^12^ (c) The application of decellularized kidney scaffolds is shown to significantly promote cell proliferation and re-endothelialization, as demonstrated in human umbilical vein endothelial cells (HUVECs), indicating their promise for supporting vascular tissue regeneration.^12^ (d) Cell sheets made of induced pluripotent stem cells (iPSC) for improving retinal pigment epithelium, the cell sheets were biocompatible and hold significant potential for treating age-related macular degeneration.^18^ (e) PEG/dextran-induced protein fibrous shells for stable encapsulation of cells at macropore interfaces through RGD peptide interactions, thereby supporting tissue development, highlight the significance of engineered cellular microenvironment.^78^ (f) Schematic overview of artificial intelligence revolutionization in the field of biomaterial design by accelerating the discovery process, reducing reliance on trial-and-error methods, and enabling faster, more cost-effective therapeutic innovations.^94^ Collectively, these advances underscore the dynamic interplay between biomaterials, cellular engineering, and computational approaches in shaping the future of regenerative medicine.

For cardiovascular applications, various cell-based technologies have been investigated for treating aortic aneurysms, creating vascular grafts, designing stents, and more. For example, bioengineered vascular grafts from human donor ovine fibroblasts implanted in 3-month-old lambs have demonstrated both recellularization and successful somatic growth capacity alongside the growing young lamb.^9^ This finding represents remarkable scientific progress in pediatric applications, where the long-term growth of implanted engineered tissues is a significant challenge. In terms of translation, “off-the-shelf” grafts grown initially with cells and later decellularized^10^ are appealing for scalability and broader applicability, given the lack of cells that obviate potential challenges like immune response in the final implanted products. For example, the avascular graft developed by Humacyte showed effective host integration aiding dialysis access and a bioresorbable conduit for improving cavopulmonary circulation [Fig. 2(a)].^11^ In another example, a decellularized scaffold from Xeltis promoted renal function and alleviated nephrectomy [Figs. 2(b) and 2(c)].^12^

Another area that has benefited from cell-based regenerative medicine is wound healing, particularly in the treatment of chronic non-healing wounds. The earliest of these therapies was marketed in the 1990s by Organogenesis and consisted of a bilayered tissue involving extracellular matrix (ECM) components, foreskin-derived neonatal fibroblasts, and foreskin-derived neonatal epidermal keratinocytes.^4^ This therapy was followed by several FDA-approved therapies and clinical trials investigating largely stem cell- and immune cell-based wound dressings.^13^ Associated complications, such as the formation of biofilms, are also being actively investigated.^13^

Stem and progenitor cells have key advantages in regenerative medicine, including the ability to restore organ function.^14^ For example, umbilical cord blood-derived hematopoietic stem cells (HSCs) and hematopoietic progenitor cells (HPCs) are among the most widely used cell types with FDA approval and success.^4^ In addition, human-induced pluripotent stem cells (iPSCs) have proven instrumental in uncovering the molecular mechanisms underlying genetic and infectious diseases, especially in cases where traditional animal models fail to replicate human pathophysiology.^15,16^ One example is the transplantation of iPSC-derived retinal pigment epithelial cells for treating age-related macular degeneration, which has demonstrated anatomical integration and early-stage visual improvement [Fig. 2(d)].^17,18^ Numerous clinical trials are currently testing the efficacy of various therapeutics to support regeneration across a variety of diseases (Table I). Together, these examples demonstrate the therapeutic promise of cell-based biologics in treating a range of diseases.

**TABLE I.** Representative examples of ongoing or recently completed clinical trials related to regenerative medicine. Abbreviation: CABG, coronary artery bypass graft; CAR-T, Chimeric antigen receptor T cell.

Type	Study title	Phase	Clinical trials registry #
Cancer			
	SHARON: A clinical trial for metastatic cancer with a BRCA or PALB2 mutation using chemotherapy and patients' own stem cells	Phase I	NCT04150042
	Anti-EGFRvIII synNotch receptor induced anti-EphA2/IL-13Ralpha2 CAR (E-SYNC) T cells	Phase I	NCT06186401
	Dendritic cell vaccination for patients with solid tumors	Phase I, II	NCT01291420
Autoimmune diseases			
	Autoimmune disease treatment with mesenchymal stem cells and CAR-T cells	Phase I, Phase II	NCT06435897
	Lymphocyte depletion and stem cell transplantation to treat severe systemic lupus erythematosus	Phase II	NCT00076752
Neurological disorders			
	Randomized double-blind placebo-controlled adaptive design trial of intrathecally administered autologous mesenchymal stem cells in multiple system atrophy	Phase II	NCT05167721
	Best available therapy versus autologous hematopoietic stem cell transplant for multiple sclerosis	Phase III	NCT04047628
Cardiovascular disease			
	Efficacy and safety of mesenchymal stem cell clusters in patients with critical limb ischemia	Phase I, Phase II	NCT04661644
	Co-transplantation of mesenchymal stem cell derived exosomes and autologous mitochondria for patients candidate for CABG surgery	Phase I, Phase II	NCT05669144

Numerous non-cell-based technologies have also found success in clinical regenerative applications. Biomaterial-based scaffolds, growth factor delivery systems, and extracellular vesicle (EV)-based therapeutics have advanced rapidly, offering complementary or alternative strategies to cell therapies. For instance, bioresorbable polymer scaffolds and decellularized matrices have demonstrated efficacy in wound repair and vascular grafting by supporting host-cell infiltration and tissue remodeling.^19,20^ Growth factor-releasing or drug-loaded hydrogels and controlled-release matrices have shown clinical benefit in promoting angiogenesis and osteogenesis, such as in diabetic ulcers and bone defect healing.^21–23^ Similarly, EVs derived from stem or immune cells are emerging as acellular biologics that recapitulate many paracrine regenerative effects without the manufacturing and storage challenges of living cells.^24^ These acellular and biomaterial-based approaches are broadening the therapeutic scope of regenerative medicine, offering scalable and standardized solutions that complement cell-based approaches in clinical translation, with many reaching clinical trials and successful commercialization in a shorter timeframe.

## REGULATORY AND TRANSLATIONAL CONSIDERATIONS

Despite key advances in regenerative medicine, scalability and large-scale manufacturing remain major challenges. The design of bioreactors must be tailored to support specific cell types while maintaining cell viability and functionality. Systems that minimize hydrodynamic shear stress, enhance oxygenation, and support the expansion of metabolically active cells are essential for clinical-scale production. For example, hollow fiber bioreactors for expanding T cells enable effective gas and nutrient exchange, allowing for the growth of billions of cells in just 10 days.^25^ Overcoming scalability issues is critical, especially for full-organ cell therapy, where several liters of cultured cells may be required. For stem cell-based therapies, maintaining the correct lineage and undifferentiated state during expansion is crucial for therapeutic success. Proper commercialization strategies, supported by quality control and process standardization, will be key to the successful clinical translation of these advanced cell therapies.

Translation of regenerative technologies depends not only on cell-intrinsic factors but also on the complexity of the product type (i.e., cellular vs acellular). Non-cell-based regenerative approaches, such as acellular scaffolds, small molecules, and EVs, may require less intensive considerations, yet maintain a critical need for quality control.^26^ These non-cellular approaches may be limited in their potential for addressing many problems where cell-based therapies cannot only be transformative but can also be creatively deployed, with generally simpler manufacturing and storage, scalability, and standardization.^26^

Regulatory challenges remain in the safety of regenerative medicine. Gene-editing-based technologies, such as CRISPR-Cas9, present concerns about unknown long-term effects following somatic interventions, as well as broader issues in cell and gene therapy applications.^27^ Regulatory frameworks such as the Regenerative Medicine Advanced Therapy (RMAT) designation in the United States and the Priority Medicines (PRIME) program by the European Medicines Agency (EMA) aim to proactively address anticipated challenges, particularly during the preclinical and clinical trial phases.^27^

In addition to these regulatory and manufacturing considerations, biological factors at the host–implant interface (i.e., wound healing and immune cell responses contributing to rejection) remain crucial. Beyond stem cells and parenchymal tissue responses, immune cell dynamics are central to regenerative outcomes. Macrophages, T cells, and other immune populations critically influence the inflammatory and remodeling phases of tissue repair. When contrasting cell to non-cell-based approaches, non-cell-based approaches have less immunogenicity;^26^ however, both face the host immune response and potential rejection as barriers to translation.^28^ While not the primary focus of this perspective, these immune-regenerative interactions, which also vary by age and sex, warrant continued attention in future studies and projects to ensure clinical safety and efficacy.^28^

Together, these translational and regulatory challenges emphasize the importance of designing therapies that are both biologically effective and manufacturable at scale, to transfer to the clinic and impact patient care.

## INTEGRATING REAL-WORLD AND MICROENVIRONMENTAL CONSIDERATIONS

To effectively address patient needs, regenerative therapies must integrate real-world considerations, such as patient demographics and the full spectrum of the lifespan. Factors such as age, sex, and disease subtype, with microenvironment optimization, shape both the fidelity of *in vitro* disease models needed for better understanding and developing these therapies and the efficacy of therapeutic interventions themselves. These parameters influence cell behavior, immune modulation, and matrix remodeling, as well as practical aspects of therapy development, including cell sourcing, manufacturing scalability, and quality control.

Despite some notable exceptions, most biologics do not progress to the stage of clinical translation. One of the important contributing factors is suboptimal integration with the cellular microenvironment. The microenvironment is composed of structural (ECM and neighboring cells) and nonstructural (physiological fluids and signaling molecules) components that support tissue function. Concomitantly, other factors, such as cell shape, cell identity, and biophysical and biochemical signals, also transduce cues to the cells that regulate cellular responses.^29^ These signals may originate from cell–cell interactions (e.g., ligand–receptor binding or cell adhesion molecules),^30^ cell–ECM interactions (e.g., bound cytokines or integrin–matrix binding),^31,32^ external physiological fluids (e.g., shear stress or osmotic pressure),^33,34^ and tissue–organ level perturbations (e.g., paracrine factors or electrical currents or movement).^29,35,36^ The dynamic reciprocal interaction between the intracellular components and the surrounding environment defines the spatiotemporal organization and function of healthy and diseased tissue.^37,38^ Furthermore, the dynamic nature of a cellular niche establishes a heterogeneous three-dimensional compartment governed by the spatiotemporal distribution of biochemical and biophysical cues, both within and outside a tissue, ultimately determining the structural development, homeostasis, remodeling, and response of a tissue.^39–41^ Therefore, understanding organ regeneration and developing tissue engineering approaches to recover or replace a cellular niche requires a close mimicry of each component of a microenvironment necessary for preserving cell identity and physiological function. Among the many factors, age and sex also represent real-world considerations as influential determinants of regenerative potential, shaping both intrinsic cellular behavior and the response to therapy.

### The impact of aging on regenerative medicine

Aging profoundly alters the cellular and molecular landscape necessary for both effectively responding to regenerative therapies and contributing viable components to autologous treatments. Evidence supports a variety of age-related changes that contribute to the accumulation of senescent cells, further exacerbating the decline in regenerative capacity with age. Senescent cells resist apoptosis while secreting pro-inflammatory factors [known as senescence-associated secretory phenotype (SASP)], creating a state of chronic, low-grade inflammation that generates a hostile microenvironment for regenerative processes and can trigger excessive fibrotic responses to therapeutic biomaterials.^42^ Similarly, age-related ECM remodeling, characterized by increased collagen cross-linking, altered glycosaminoglycan profiles, and modified integrin–matrix interactions, restricts cellular migration, impairs mechanotransduction, and disrupts tissue architecture.^43–45^ Collectively, these age-associated changes manifest clinically as a reduced efficacy of regenerative therapeutics. Autologous cell-based approaches are particularly affected, given that donor cells exhibit age-related functional decline. These studies underscore age as a critical factor in regenerative medicine.

Novel approaches targeting the aging phenotype hold promise for enhancing regenerative outcomes. A multitude of senolytics—compounds that selectively clear senescent cells—have been shown to rejuvenate the tissue microenvironment by reducing senescent cell burden and mitigating SASP-related inflammation^46^ to improve osteoarthritis,^47^ sarcopenia,^48^ and fracture healing.^49^ Additionally, recent advances in epigenetic reprogramming demonstrate partial rejuvenation of aged cells. Techniques employing modified Yamanaka factors^50^ or targeted epigenetic editors can reset age-associated deoxyribonucleic acid (DNA) methylation patterns and chromatin accessibility, effectively reversing cellular biological age while preserving the differentiated state critical for tissue function.^51,52^ These strategies may help slow down the biological aging process, with promise for improving the reliability and translational readiness of regenerative therapies across diverse patient populations.

### Sex differences in regenerative medicine

Regenerative medicine seeks to modulate the body's intrinsic response to injury by targeting key processes, including inflammation, cell differentiation and migration, and mechanical stimuli. These programs show profound sex differences across tissues, with contributions from both genetic karyotype and exposure to sex hormones. However, few studies have investigated the effect of sex on biomaterials. Recent reports have shown sexual dimorphism in the endothelial cell response to engineered scaffolds and mechanical stimulation.^53^ Male and female cells were reported to remodel the local ECM differently, which may lead to sex differences in functional outcomes after biomaterial implant.^53–55^ Additionally, preclinical studies have reported lower numbers of mesenchymal stem cells (MSCs) in female rats, which negatively impacts bone healing.^56^ However, MSCs from female donors maintain proliferative capacity longer *in vitro*, making them more attractive for use in engineered tissues and potentially more therapeutically beneficial in regenerative studies than male MSCs.^57^ There are also differences in both innate immune mechanisms and functional immune cell responses, which could affect the efficacy of regenerative therapies, such as discussed for adeno-associated virus gene therapies,^58^ and require further investigation. Recent technological developments in biomaterials and multi-omics computational methods may advance our understanding of sex as a biological variable in regenerative medicine.^59^ As these therapies continue to emerge, sex differences will be important to understand and tailor these treatments to the patient populations they serve, moving toward precision medicine.

### Integrating multiple cues

Conventional culture systems, which mimic a single component of the cellular microenvironment, are advantageous when studying the intrinsic mechanisms of a morphological or functional trait.^60–62^ However, they often lack the cellular complexity observed *in vivo* and fail to adapt to constantly changing cues. In contrast, systems that model two or more types of microenvironmental factors better mimic native cellular niches. For example, some technologies incorporate fluid flow within engineered 3D organotypic models^63^ or employ microfluidics^64^ to model the effect of interstitial and luminal fluid pressure on vascular and lymphatic circulatory systems. Despite recent efforts to integrate multiple microenvironmental cues, the mechanisms by which multi-factorial external cues elicit specific cellular responses, along with the integration of various cues to recapitulate biologically relevant responses, remain poorly understood. Considering the challenges associated with manufacturing technologies and understudied physiological cues, current approaches aiming to mimic cellular microenvironments prioritize essential cues for tissue function, with the underlying assumption that a more realistic niche encourages cellular components to recover or maintain their native states. Addressing these knowledge gaps will enhance our ability to reconstruct functional tissues and develop therapies that more closely resemble specific microenvironments.

### Spatiotemporal regulation of external cues

One salient consideration of external cues is their spatiotemporal organization to recapitulate the structural architecture and function of a tissue as an interconnected composite of various cells and ECMs. To recapitulate tissue formation *in vitro*, engineered tissues may be fabricated as a standalone construct or integrated into cell culture platforms (i.e., microfluidic chips and templating molds). Recently, spatial control during biofabrication has been advanced by the filamented-light fabrication technique,^65^ the hydrogel localization platform,^66^ and the high-resolution metallic sacrificial molding technique.^67^ In particular, there is an increasingly sophisticated development of 3D bioprinting techniques for standalone construct biofabrication.^68–72^ However, biofabricating a macroscale-sized tissue with microscale resolution remains a major challenge that limits the scalability of spatial control. While volumetric bioprinting shows the most potential to surpass this limitation, the technique itself is currently applicable using one bioink type at a time, which impedes the ability to recapitulate the spatial heterogeneity of ECM in an engineered tissue.^73,74^ Furthermore, leveraging the spatial heterogeneity of hydrogels in biofabrication provides the means to localize bioactive molecules via covalent conjugation and/or noncovalent affinity (i.e., heparin-binding affinity,^75^ biotin–streptavidin interaction,^76^ and affibody molecules^77^) Another example is the use of biomaterial-based cell encapsulation consisting of an engineered macropore interface for optimal cellular function [Fig. 2(e)].^78^ Accordingly, breaking through the spatial limit of size scale, resolution, and heterogeneity remains a challenge for optimizing the environment at a tissue level.

Although spatial control during biofabrication can optimize the environment to stimulate tissue formation, temporal control is important for tissue maturation and disease progression. While some engineered tissues can reach homeostasis or functional maturation based on cellular dynamics, a timely exposure or actuation is often required to guide tissue development *in vitro*. Temporal exposure to biochemical cues (e.g., growth factor and cytokine chemical inhibitors) is a well-established strategy to guide the differentiation and organization of organoid tissues,^79^ and sequence-specific biochemical cues can impact tissue regeneration.^80^ Similarly, periodic mechanical stimulation is a major driver of skeletal myotube alignment, resulting in the development of functional and mature muscle tissue.^81,82^ In particular, photochemistry is a powerful tool for both spatial and temporal control of ECM properties,^83,84^ enabling applications in both *in vivo* and *ex vivo* settings.^85^ An automatic transitioning system that guides tissue formation to a terminally differentiated and mature state is the next challenge in temporal control for environmental optimization.

For *in vivo* applications, the type of graft (i.e., acellular scaffolds vs cellular engineered tissues) dictates the parameter considerations for spatiotemporal environmental optimization. To promote host-tissue regeneration in an acellular scaffold, the scaffold's degradation should be temporally controlled to match the speed of host tissue ingrowth.^86^ Moreover, spatiotemporal control of bioactive molecules or cytokines in a scaffold with drug-delivery or controlled-release systems can further stimulate host tissue regeneration.^87,88^ On the contrary, optimally integrated cell-based engineered tissues should be able to withstand degradation as well as remodel over time,^89^ in addition to implementing spatiotemporal controls during *in vitro* biofabrication. Accordingly, a source of tissue vascularization must be considered for long-term survival of grafted tissue.^90–92^ Together, the spatial and temporal control of the graft environment, as well as the host environment, are crucial for successful tissue engineering and regeneration.

## COMPUTATIONAL ADVANCES IN REGENERATIVE MEDICINE

Computational approaches in regenerative medicine have accelerated with unprecedented opportunities to optimize complex biological systems and treatments. As regenerative medicine evolves, the integration of artificial intelligence, multiscale modeling, and data analytics has enhanced mechanistic understanding while guiding clinical translation.

### Mechanistic and Multiscale Modeling

The integration of computational modeling with experimental approaches has become integral to advancing the field of Tissue Engineering and Regenerative Medicine (TERM).^93^ Mechanistic models, grounded in experimental and theoretical insights, globalize domain-specific, molecular events to explain causality and biological systems. These models integrate molecular, cellular, and tissue phenomena to simulate or predict regenerative mechanisms. Network models of signal transduction pathways, such as the Hippo or WNT pathways, inform predictions of cell growth, proliferation, and morphogenesis. From collagen remodeling in engineered cardiovascular tissues to simulating scaffold compliance, computational models can analyze microenvironmental responses, simulate specific behavioral patterns, and inform experimental design.^93^

Multi-omics approaches provide further mechanistic insights into regenerative medicine by characterizing cellular identities and interactions within tissues. In particular, single-cell RNA sequencing (scRNA-seq) enables researchers to capture specific molecular signatures, cell states, and dynamic responses to injury, aging, or biomaterials.^94^ The integration of scRNA-seq data with other modalities, such as scATAC-seq or imaging data, would further enhance the characterization of cellular subtypes and the prediction of communication networks integral to regenerative therapies.^95^ These approaches contribute to strategies that target both transcriptional regulators and their upstream signaling pathways, accelerating the development of precision-engineered therapies in clinical translation.

### Stem cell computational modeling

Recent computational advances have transformed our understanding of stem cell work. A study developed mechanistic computational models to analyze variations in stem cell graft characteristics and their influences on allogeneic HSC transplantation (all-HSCT).^96^ The model examines how transplant dose affects clonal dynamics and incorporates multiple cell types, including parameters such as proliferation rate and self-renewal probability.^96^ The study's computational approach reveals how identical recipients respond to different transplant doses. This finding would be challenging to replicate in clinical settings where patients can only receive one transplant at a time. Beyond hematopoietic applications, computational models are also used to study other areas, such as neural tissue development. The Biochemical Neural Network Model framework simulates neural tissue development from progenitor cells that differentiate into specialized neural cells.^97^ In the presented computational models, neuronal connectivity displayed patterns similar to real structures, enabling researchers to explore regulatory factors in these processes. These examples highlight the utility of predictive computational models that can surpass the pace of research findings derived from basic science alone.

### Machine learning (ML) and artificial intelligence (AI) in regenerative medicine

Perhaps the most notable advances within predictive modeling are driven by AI. Some of the most promising applications of AI lie in *in silico* simulation of tissue responses to dynamic stimuli, which allows researchers to optimize testing. Computational algorithms can simulate scaffold–tissue interactions and predict cell migration, proliferation, and differentiation. ML pipelines predict mechanical strength, cell adhesion, porosity, and many more biomaterial properties. Support Vector Machines, Random Forests, and other supervised ML algorithms demonstrated strong performance in predicting features of scaffolding based on composition and structure.^98^

Beyond biomaterial properties, deep learning architectures, convolutional neural networks, and graph neural networks can be leveraged in current workflows to predict cellular and molecular behavior in particular environments. These models capture nonlinear relationships and spatial scales, answering pre-experimental questions. Another important area of application for deep learning algorithms is the analysis of multi-omics data (i.e., transcriptomics, proteomics, and metabolomics) to generate mechanistic hypotheses for patterning cellular behaviors [Fig. 2(f)].^94^

Linking these AI models to clinical data, a continuous feedback loop between computational predictions and real-world outcomes is established. ML pipelines work to combine omics, imaging, and patient data to generate models that are fine-tuned through empirical validation. For example, neural networks have successfully predicted regenerative abilities in vascular and tendon tissue engineering while matching these findings with experimental data.^99^ Methods to standardize data formats, such as integrating laboratory data in standard Digital Imaging and Communications in Medicine (DICOM) or Picture Archiving and Communication Systems (PACS) imaging file formats, enhance reproducibility and further validation.^99^ The integration of fine-tuned ML models with robust experimental and clinical data strengthens interpretability and accelerates translation of computational models into regenerative medicine strategies.

The personalization of regenerative therapies has long been a key and extensively studied area within precision medicine. Computational regenerative medicine with the use of ML models can enable personalized and patient-specific therapies. From scaffold design to therapeutics, ML can generate recommendations and perform analyses through individual patient data, such as genetic profiles. In the context of drug discovery, AI-based regenerative medicine focuses on network biology, where machine learning simulates complex interaction networks, including drug–gene interactions, signaling pathways, crosstalk, and protein–protein interactions. These models are crucial in pointing out bottlenecks within regenerative medicine, such as inhibitory or inflammatory pathways or transcriptional blockages on the cellular level. ML algorithms, such as Bayesian network optimization and deep neural networks, can resemble pharmacokinetics and expected outcomes for therapeutics. The standardization of ML models in the future will likely improve their adoption into mainstream research workflows and better inform research and translation of therapeutic agents.

## CONCLUSION

In summary, regenerative medicine rests at the intersection of engineering, biology, and medicine, offering unprecedented potential to restore and repair tissue and organ function. This perspective has explored the technical, biological, and translational advances shaping the field, from optimizing cell and tissue microenvironments to integrating sex and age as critical biological variables, to overcoming scalability barriers, and to applying computational tools that guide design and discovery. These technological advances are likely to increase the number of regenerative therapies that successfully advance to clinical translation and have an impact on patient health across various diseases.

## Data Availability

Data sharing is not applicable to this article as no new datasets were generated or analyzed in this work. All data discussed in this review are available within the published literature cited in the reference list.
